# Isolation and Identification of Alkaloid Genes from the Biomass of *Fritillaria taipaiensis* P.Y. Li

**DOI:** 10.3390/metabo14110590

**Published:** 2024-10-31

**Authors:** Nong Zhou, Chun-Mei Mei, Fu-Gui Chen, Yu-Wei Zhao, Ming-Guo Ma, Wei-Dong Li

**Affiliations:** 1 College of Pharmacy, Nanjing University of Chinese Medicine, Nanjing 210023, China; 20120038@sanxiau.edu.cn (N.Z.); 20210695@njucm.edu.cn (C.-M.M.); 20210690@njucm.edu.cn (F.-G.C.); 20221018@njucm.edu.cn (Y.-W.Z.); 2 College of Food and Biological Engineering, Chongqing Three Gorges University, Chongqing 404120, China; 3 College of Materials and Science and Technology, Beijing Forestry University, Beijing 100083, China

**Keywords:** biomass, alkaloid genes, identification, metabolic group, transcriptome

## Abstract

Background/Objectives: *Fritillaria taipaiensis* P.Y. Li is a valuable traditional Chinese medicinal herb that utilizes bulbs as medicine, which contain multiple alkaloids. Biomass, as a sustainable resource, has promising applications in energy, environmental, and biomedical fields. Recently, the biosynthesis and regulatory mechanisms of the main biomass components of biomass have become a prominent research topic. Methods: In this article, we explored the differences in the heterosteroidal alkaloid components of *F. taipaiensis* biomass using liquid chromatography–mass spectrometry and high-throughput transcriptome sequencing. Results: The experimental results demonstrated significant differences in the eight types of heterosteroidal alkaloid components among the biomass of *F. taipaiensis*, including peimisine, imperialine, peimine, peiminine, ebeinone, ebeiedine, ebeiedinone, and forticine. Transcriptomic analysis revealed substantial significant differences in gene expression patterns in the various samples. Three catalytic enzyme-coding genes, 3-hydroxy-3-methylglutaryl coenzyme A synthase (*HMGS*), 3-hydroxy-3-methylglutaryl coenzyme A reductase (*HMGR*), and terpene synthase (*TPS*), were speculated to contribute to the regulation of the differential accumulation of alkaloid synthesis in *F. taipaiensis* bulbs. A strong positive correlation was observed between the transcriptional level of the *TPS* gene and the alkaloid content of *F. taipaiensis* biomass, suggesting that *TPS* may be a key gene in the biosynthesis pathway of alkaloids. This finding can be used for subsequent gene function verification and molecular regulatory network analysis. Conclusions: This work provides fundamental data and novel insights for the subsequent research on alkaloid biosynthesis in *F. taipaiensis*.

## 1. Introduction

Biomass is a sustainable resource, that has wide applications in the energy, environment, and biomedical fields [1,2,3]. Currently, research has focused on the separation, component analysis, and active alkaloid ingredients of biomass [4]. In particular, the biosynthesis and regulatory mechanisms of the main active components of biomass have garnered increasing attention [5,6]. Recently, transcriptome sequencing has emerged as a promising strategy for exploring gene expression regulation [7,8]. Transcriptomes can reflect the specific expression of biomass genes at a specific time and space, effectively demonstrating the differential expression of genes after the introduction of biomass to different regions, resulting in inconsistent secondary metabolites and variations in effective ingredients [9]. For instance, Zhao et al. reported transcriptome analysis and plotted the gene expression profile of *Fritillaria cirrhosa* [10], which is a valuable traditional Chinese medicinal herb that uses bulbs as medicine and is listed in the Pharmacopoeia of the People’s Republic of China under the name of “ChuanBeiMu” [11].

Heterosteroidal alkaloids are a class of alkaloids characterized by a C_27_ skeleton system, comprising five to six carbon rings in their basic skeleton structure. Their distinctive ring structure is the C-nor-D-homo-[14(13→12)-abeo] ring system, which includes the unit of the peritendinous ring [4]. Heterosteroidal alkaloids are mainly found in plants of the Liliaceae family, specifically within the genera *Fritillaria* and *Veratrum*, and are the primary components responsible for the pharmacological activity in these two plant genera [12]. Li et al. reported a transcriptome analysis of the cytochrome P450 oxidase (FcCYP) gene family in *F. cirrhosa*, indicating a positive correlation between FcCYP transcriptional expression and the accumulation of steroid alkaloids [13]. However, limited studies focus on the components and biosynthesis mechanisms of alkaloids in biomass [14,15]. In particular, the gene function of alkaloid biosynthesis and the regulatory mechanism of transcription factors in biomass remain unclear [16]. *Fritillaria taipaiensis* P.Y. Li is a perennial herb in the Liliaceae family, primarily distributed in southwestern China [17]. Its bulbs contain multiple alkaloids that can cool down body temperature, relieve coughing and asthma, and resolve phlegm; it is also listed in the Pharmacopoeia of the People’s Republic of China as “ChuanBeiMu” [18].

Herein, in order to explore the alkaloid biosynthesis key gene and its regulation mechanism in *F. taipaiensis*, the alkaloid and differentially accumulated metabolite content in the *F. taipaiensis* bulb from different regions was determined and the differentially expressed genes (DEGs) in the biomass of *F. taipaiensis* were identified using integrated transcriptomics technology. This study will contribute to improving the quality of biomass by transcriptomics regulation.

## 2. Materials and Methods

### 2.1. Samples

Based on a preliminary investigation by the research group, the geographical distribution and ecological differences in specimens led to the determination of representative sampling points for biomass *F. taipaiensis* (including wild species). In July 2022, >300 3-year-old biomass *F. taipaiensis* plants were randomly excavated using the snake-shaped method and each collection point was mixed at multiple points. Bulbs of biomass of *F. taipaiensis* plants were collected as samples. Samples from each production area were biologically replicated three times, washed with water, immediately frozen in liquid nitrogen, and divided into two parts. One part was stored at −80 °C for total RNA extraction while the other part was dried by hot air at a temperature of 50–55 °C for metabolite extraction. Information and codes regarding the origins of biomass *F. taipaiensis* from different regions are shown in Table 1. The soil property information is presented in Table S1. The experimental samples were subjected to high-throughput sequencing analysis by Beijing Novogene Technology Co., Ltd. (Beijing, China). Three independent biological replicates were used for each sample.

### 2.2. Metabolomics Study

Approximately 1.0 g of biomass of *F. taipaiensis* powder (sieved through a 200-mesh sieve) was selected. This powder was added to 25 mL of 60% ethanol, soaked for 20 min, and refluxed at 90 °C for 1 h for extraction. These steps were repeated twice. The continuous filtrate (1 mL) was accurately measured and passed through a SPF column, which was then diluted and eluted with different ratios of methanol in a gradient ranging from low to high concentrations. The diluted sample was then centrifuged at 12,000 r.min^−1^ for 10 min, after which the supernatant was pipetted, and it was passed through a 0.22 μm filter membrane [19].

Mass spectrometry (MS) of the samples was performed in both positive and negative ion modes using a dynamic background subtraction function with a scanning time of 840 ms, equipped with an automatic calibration delivery system. The chromatographic conditions of the samples were determined by using an Agilent Zorbax Extended C18 UHPLC Column (Agilent, Beijing, China).

### 2.3. Transcriptomics Study

RNA integrity and total RNA were accurately assessed using an Agilent 2100 Bioanalyzer (Agilent, Beijing, China). Total RNA that met the standards was utilized for library construction. After passing the quality test, the library was subjected to high-throughput sequencing using an Illumina NovaSeq 6000 (Illumina, San Diego, CA, USA) by Beijing Novogene Technology Co., Ltd (Beijing, China).

New gene predictions were made using StringTie (version 1.3.3b). This software utilized the official National Center for Biotechnology Information Protein Sequence database (RefSeq non-redundant proteins (NR)), Nucleoside Sequence (NT) database, Kyoto Encyclopedia of Genes and Genomes (KEGG), SwissProt Protein Quality database (manually annotated and reviewed protein sequence database), Protein Family (PFAM) database, Gene Ontology (GO) database, and Eukaryotic Protein Homologous Groups (KOG) database to annotate the gene functions of the obtained transcripts. DESeq2 (1.20.0) was employed for intergroup differential expression analyses.

To verify the DEGs identified, three candidate genes (3-hydroxy-3-methylglutaryl coenzyme A synthase (*HMGS*), 3-hydroxy-3-methylglutaryl coenzyme A reductase (*HMGR*), and terpene synthase (*TPS*) were selected for quantitative reverse transcription PCR (qRT-PCR). First, 2 μg of total RNA was converted to complementary DNA (cDNA) using a one-step gDNA removal and cDNA synthesis kit (TransGene, Beijing, China). After diluting the samples five times, real-time fluorescence quantitative PCR was performed using an Eppendorf MasterCycle PCR instrument (Eppendorf, Hamburg, Germany). The *rpl16* gene (F: TTCGTGCTACATTCGTAGGGGGTC; R: GTTCCATTGCGGAGTTCGG) of the biomass of *F. taipaiensis* was used as an internal reference gene and each sample was analyzed in triplicate. Primers were designed using Primer Premier 6.0 and Beacon Designer 7.8 software (Table 2) and the relative expression of the gene was calculated using the 2^−ΔΔCt^ method. The PCR products were analyzed and purified by agarose gel electrophoresis and sequenced by Nanjing Aoqing Biotechnology Co., Ltd. [20].

### 2.4. Data Analysis

The data are presented as mean ± standard deviation, the data were analyzed by a single factor (one-way ANOVA) for significance analysis via SPSS 25.0 and Excel 2003 (α = 0.05), and correlation analysis was conducted on SPSS 25.0 using the Pearson method (α = 0.05). The QIIME software (version 1.9.1) was used to calculate the Unifrac distance. Principal component analysis (PCA) was performed by vegan bag on R-software (version 4.3.2). GraphPad Prism 8 and R-software (version 4.3.2) were used for graphics analysis and Adobe Illustrator (version 28.1.0) was used for graphic processing.

## 3. Results

### 3.1. Chemical Constituents and Differential Metabolites

Potential compounds were inferred by analyzing the tandem mass spectrometry (MS/MS) fragmentation patterns of the substances (Table S2). In total, 71 compounds were identified in the positive ion mode, 34 compounds were identified in the negative ion mode, and 83 compounds were identified in both positive and negative ion modes. This included thirty alkaloids (Compounds **1**–**2**, **4**–**5**, **8**–**18**, **22**–**36**), six alkaloid glycosides (Compounds **3**, **6**–**7**, **19**–**21**), two fatty amides (Compounds 37–38), nine nucleosides (Compounds **39**–**46**, **48**), one peptide (Compound **47**), six fatty acids (Compounds **49**–**53**, **59**), six aromatic acids (Compounds **54**–**58**, **60**), ten lipids (Compounds **61**, **63**, **65**, **69**, **71**–**73**, **75**–**77**), one acid anhydride (Compound **62**), three aromatic aldehydes (Compounds **64**, **66**, **74**), one aromatic alcohol (Compound **67**), one aromatic ketone (Compound **68**), one phenol (Compound **70**), one fatty alcohol (Compound **78**), and five sugar components (Compounds **79**–**83**).

**Figure 1:**
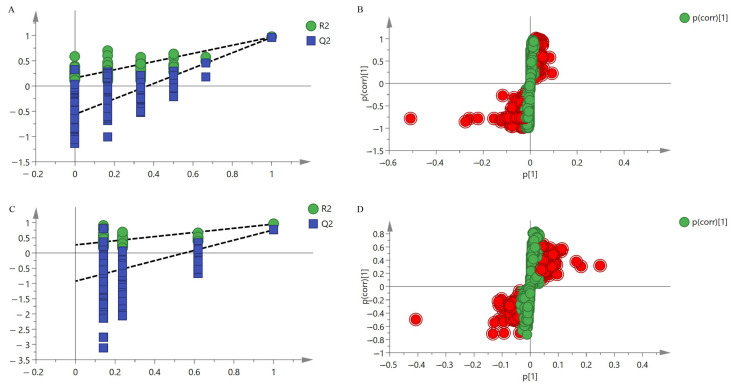
Plot of the OPLS-DA permutation test and S-plot scores for different biomass samples. (**A**) Permutation test in positive ion mode; (**B**) S-plot diagram in positive ion mode; (**C**) Permutation test in negative ion mode; (**D**) S-plot diagram in negative ion mode.

To clarify the differences in chemical compositions among different biomass samples, an orthogonal partial least squares discriminant analysis (OPLS-DA) model was developed (Figure 1). The OPLS-DA model established in both positive and negative ion modes yielded good prediction results. All simulated R^2^ and Q^2^ values on the left were less than the true values on the right, demonstrating reliable model validation (Figure 1A,B). The model identified a total of thirty-eight differential components in the positive ion mode and two differential components in the negative ion mode, totaling thirty-eight differential components (Figure 1C,D). These differential metabolites are mainly steroid alkaloids, including peimine, songbeisine, songbeinine, imperialine, delafrinone, peimenine, ebeinone, isodelavine, isoforticine, petilidine, puqiedine, ebeinine, delavine chuanbeinone, edebedi-none, puqiedinone, songbeinone, peimisine-3-O-β-D-glucopyranoside, isoverticine, delafrine, forticine, sipeimine-3-O-β-D-glucoside, isoverticine-β-N-oxide, yibeinoside B, yibeinoside A, puqiedinone-3-O-β-D-glucopyranoside, imperialine-β-N-oxide, cirrhosinine B, solanidine, cis-13 docosenoamide, 1,6-caprolactam, glyceryl monostearate, 1-monolinolein, linolenic acid, linoleic acid, and palmitic acid.

**Figure 2:**
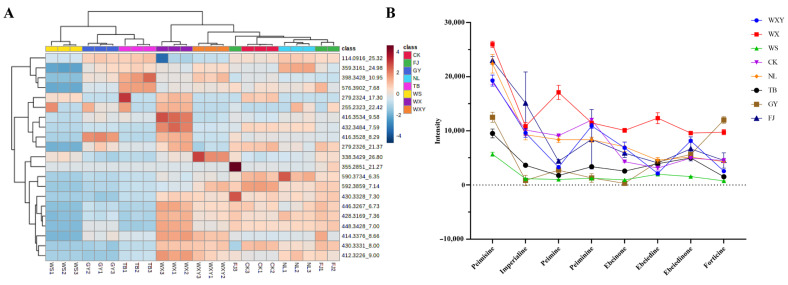
Results of semi-quantitative analysis of differential metabolites in different biomass samples. (**A**) Differential metabolite heat map with the classification tree of metabolites on the left and the classification tree of samples above; (**B**) Differential metabolite line graph.

The relative content of the discrepant components (components sharing the same mass-to-charge ratio/retention time might be isomers) was analyzed. As depicted in Figure 2A, disparities exist in the relative content of each component among different origins and the content variations are conspicuously distinct. A clustering tendency emerges among the different components across different origins. Eight types of heterosteroidal alkaloids with significant differences among the different biomass samples were selected: peimisine, imperialine, peimine, peiminine, ebeinone, ebeiedine, ebeiedinone, and forticine. The response values of these eight significantly different indicator components from different biomass samples were statistically analyzed. As presented in Figure 2B, the contents of peimisine, imperialine, peiminine, ebeinone, and ebeiedinone were relatively high in the samples of WXY, WX, WS, NL, and FJ and were low in the other samples. The peimine was highest in the sample of WX, followed by the samples of CK and NL, and was relatively low in the other samples. Ebeiedine content was highest in WX and lower in the other samples. Forticine was relatively high in two samples of WX and GY, whereas it was relatively low in the other samples. These results further indicate that the differential components represent significant differences in abundance, demonstrating the chemical diversity of different biomass samples.

### 3.2. Transcriptome High-Throughput Sequencing Analysis

To further investigate the key genes affecting the accumulation of alkaloids in different biomass samples, cultivated species from seven habitats were collected (with three biological replicates in each group) and sequencing libraries were constructed for transcriptome sequencing (Table S3). Using Trinity software (version 2.15.1), high-quality sequences obtained after quality control were assembled, resulting in 359,529 transcripts and 174,827 unigenes (Figure S1). These results indicate high transcriptome sequencing and assembly integrity, which can be used for subsequent annotation analysis.

**Figure 3:**
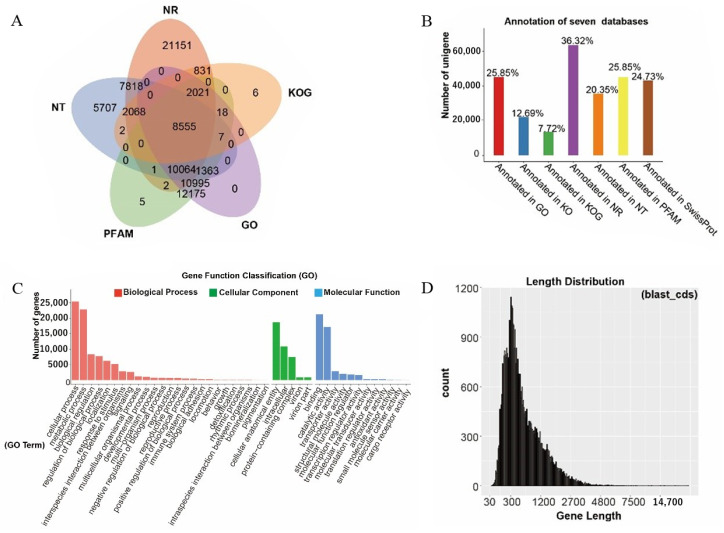
Transcript coding gene annotation. (**A**) Venn diagrams annotated in five databases (NR, NT, KOG, PAMF, GO). (**B**) Histogram of the proportion of annotatable unigenes in the seven databases. (**C**) Annotatable unigene GO clustering analysis. (**D**) Annotatable unigene length distribution map.

We compared all the unigenes obtained through transcriptional assembly using different protein databases to predict functional genes. The results showed that the highest number of unigenes (63,506) was annotated in the NR database, whereas the lowest number of unigenes was annotated in the KOG database, with 13,508 (Figure 3A). The numbers of unigenes annotated in the other databases were NT (35,586), KO (22,200), SwissProt (43,241), PFAM (45,206), and GO (45,198). The proportions of annotated unigenes in the seven databases were consistent with the numbers of annotated unigenes. Except for the comparison between the KOG and KO databases, where the content of annotated unigenes was <20%, the percentages of annotated unigenes in the remaining five databases were NR (36.32%), NT (20.35%), SwissProt (24.73%), PFAM (25.85%), and GO (25.85%; Figure 3B). The annotated unigenes were further enriched to clarify their biological functions. GO functional enrichment analysis demonstrated that the mainly annotated unigenes pertained to biological processes, such as cellular processes, metabolic processes, biological regulation, localization, and response to stimuli. Unigenes are mainly members of cellular components, such as cellular anatomical entities and intercellular and protein-containing complexes. From a molecular functional perspective, the main functions of the unigenes include binding, catalytic, and transporter activities (Figure 3C). The transcriptome sequencing results showed that the length of the transcripts varied between 100 and 3000 bp, with an average transcript length of approximately 500 bp, which could be used for subsequent analyses (Figure 3D).

**Figure 4:**
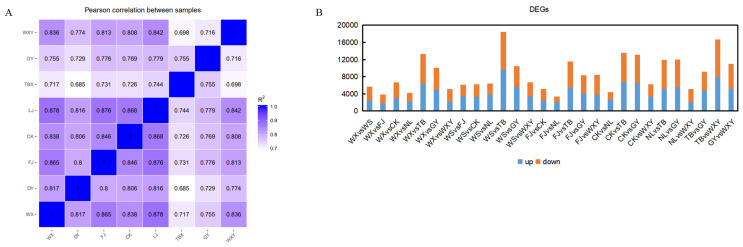
Differences in the number of transcripts in the biomass from different biomass samples. (**A**) Squared Pearson’s correlation coefficients of transcripts from different biomass samples. (**B**) Number of differentially expressed genes in different biomass samples.

The square of the Pearson correlation coefficient (R^2^) between samples was developed to represent the correlation between RNA samples from different biomass regions. The results showed that the correlation between each group of samples ranged from 0.685 to 0.878, with the maximum difference observed being between samples from WS and TB (0.685), whereas samples from WX and NL exhibited the highest correlation (0.878; Figure 4A). We further analyzed the number of DEGs in each sample, which was consistent with Pearson’s correlation coefficient. The WS sample had the highest number of DEGs compared with the TB sample, totaling 18,407 DEGs, of which 9710 genes were upregulated and 8697 genes were downregulated. Notably, samples from the TB showed the most significant differences compared to samples from other groups, with a total of >10,000 DEGs compared to each group. The number of DEGs between samples from the GY and samples from other groups displayed the second-highest difference, totaling over 8000 (Figure 4B). The FJ sample displayed a lower change in its DEG number in comparison to the other samples. The DEG number of the WXY group, was higher than that of the groups WX and NL, whereas the samples of groups TB and GY showed greater differences.

**Figure 5:**
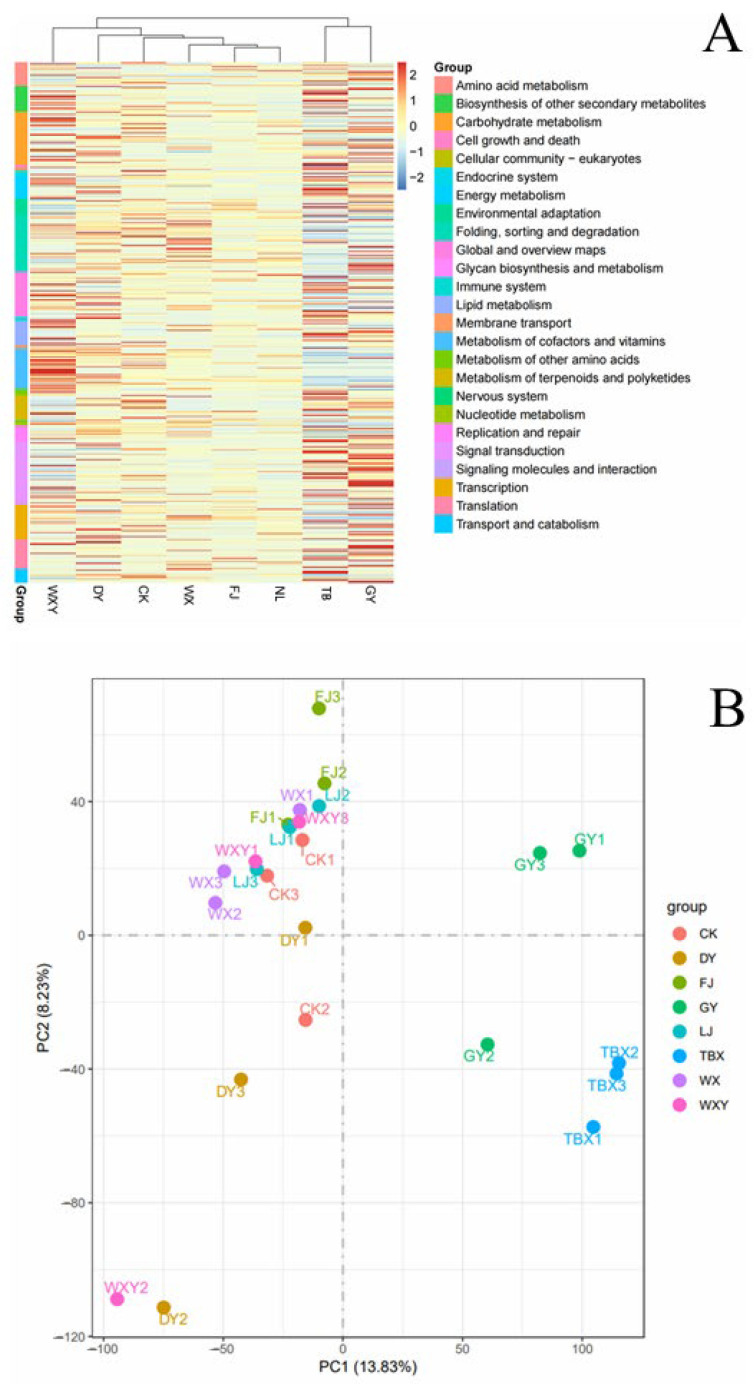
Differential gene clustering and principal component analysis. (**A**) Heat map of differential gene clustering. (**B**) Differential gene principal component analysis plot.

To further analyze the differences in gene expression between samples, an integrated analysis of differentially expressed genes (DEGs) across various groups and principal component analysis (PCA) were conducted. The number of DEGs was consistent with the observed differences in gene expression. The heat map of DEG clustering indicated that samples of groups NL, FJ, WX, CK, WS, and WXY were clustered into one branch, with the similarity between samples from different cultivation groups being greater than that between them and WXY. In WXY, the expression levels of various genes were more balanced compared with the other samples in the same branch and the expression levels of genes related to cell growth and death; biosynthesis of other secondary metabolites; and metabolisms of carbohydrate, lipid, cofactors, and vitamins were relatively high. In NL and FJ, the expression levels of most genes were relatively low compared with the other groups in the same branch (Figure 5A). The TB and GY groups were clustered separately into a single branch, with the higher expression levels of the genes related to the signal transduction, replication, and repair compared with the other groups, exhibiting different expression patterns compared to the WXY sample (Figure 5A). PCA revealed that samples from WXY, the four samples of WX, CK, FJ, and WS, and NL were clustered together, whereas those of the GY and TB samples were clustered separately; this is consistent with the findings of the DEG number and clustering analysis (Figure 5B). In summary, gene expression in different biomass samples is influenced by various conditions.

**Figure 6:**
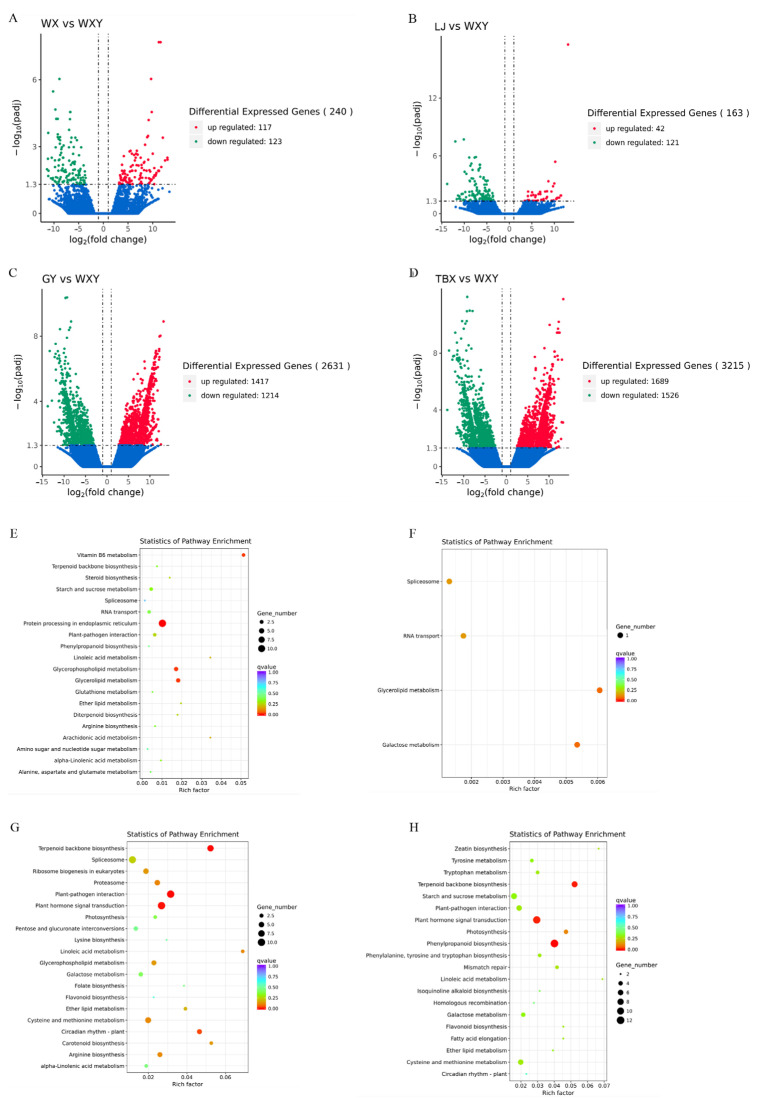
Differential gene expression and KEGG enrichment analysis between wild and cultivated cultivars. (**A**–**D**) Volcano map of differentially expressed genes between wild (WXY) and cultivated samples from (**A**) WX, (**B**) LJ, (**C**) GY, and (**D**) TB. (**E**–**H**) The differentially expressed gene KEEG-rich in the wild sample (WXY) and cultivated samples from (**E**) WX, (**F**) LJ, (**G**) GY, and (**H**) TB.

Differences in gene expression between cultivated and wild species in different biomass samples were analyzed using the WX sample as a representative of cultivated varieties. The results showed 240 DEGs (117 upregulated genes and 123 downregulated genes) in WXY and WX (Figure 6A), with the difference between these samples and the NL being primarily attributed to 163 DEGs (42 upregulated and 121 downregulated genes; Figure 6B). However, a higher DEG number was provided from the samples of WXY, GY, and TB. Specifically, the number of DEGs between the WXY and GY samples was 2631 (1417 upregulated genes and 1214 downregulated genes; Figure 6C), whereas the number of DEGs between the WXY and TB samples was 3215 (1689 upregulated and 1526 downregulated genes; Figure 6D), nearly 10 times the number of DEGs derived from the WXY, WX, and NL samples. The number of DEGs varied greatly among the different samples, indicating that the gene regulation of the WX and NL samples was more similar to that of wild species, whereas the gene regulation of the TB and GY samples was similar to that of cultivated species.

The KEGG pathway enrichment analysis of DEGs from the above four different samples showed that the DEGs in WXY and WX were concentrated in pathways such as protein processing in the endoplasmic reticulum, glycerol phospholipid metabolism, and glycerol lipid metabolism (Figure 6E). There were no significant differences in the metabolic pathways compared with NL (Figure 6F). In contrast, the DEGs of the WXY, GY, and TB samples were enriched in the biosynthesis of the terpenoid backbone, a vital step in alkaloid synthesis, suggesting that the samples of WXY, GY, and TB have different genes regulating alkaloid biosynthesis than those of the WXY, GY, and TB samples. This finding may be directly related to the alkaloid content of the sample region. Differential genes associated with the plant hormone signal transduction pathway were enriched in the comparisons of WXY vs. TB and WXY vs. GY samples, suggesting that their differences in quality may be due to hormones. Additionally, genes involved in phenylpropanoid biosynthesis were differentially enriched in the samples of WXY and TB, whereas genes involved in the plant hormone signal transduction pathway were differentially enriched in the samples of WXY and GY. Genes related to plant–pathogen interactions and circadian rhythms were enriched in the WXY samples compared to their levels in the GY sample.

### 3.3. Genes Related to the Biosynthesis of Alkaloids in Biomass

**Figure 7:**
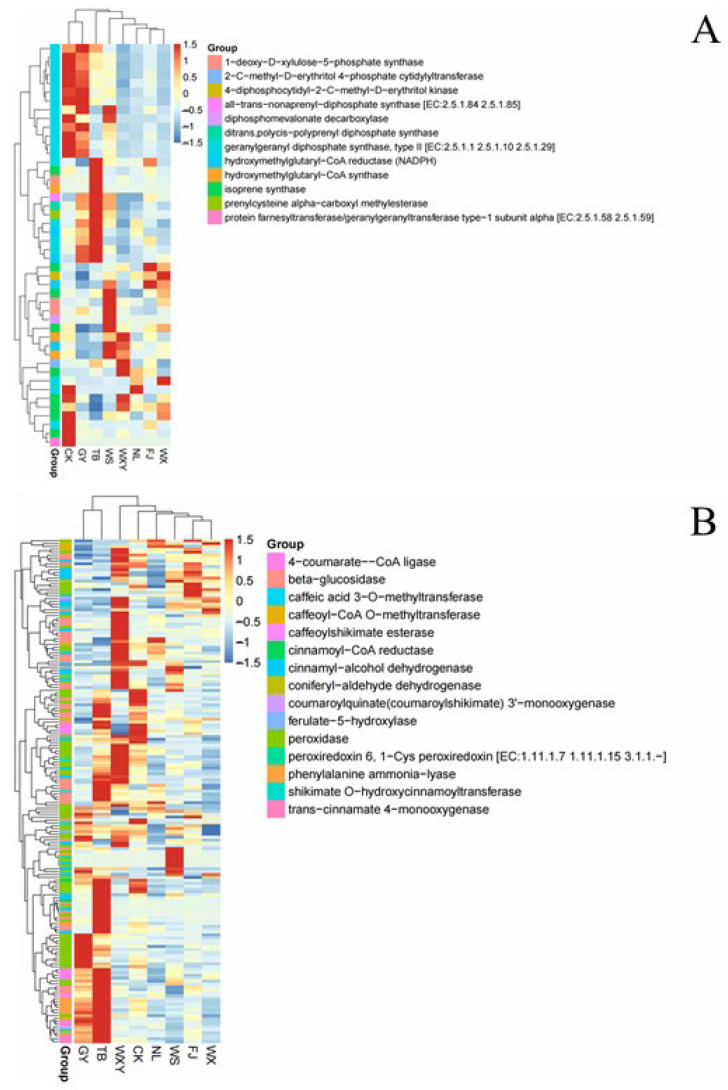
Expression analysis of key genes for the biosynthesis of active ingredients in biomass. (**A**) Heat map of differential gene clustering in terpene skeleton biosynthetic pathways. (**B**) Heat map of differential gene clustering in the phenylpropanoid biosynthetic pathway.

Based on the GO enrichment analysis of DEGs, we found that the DEGs in the “terpenoid skeleton biosynthesis” and “phenylpropaneoid biosynthesis” pathways impacted the various biomass samples. To further clarify the formation mechanism and identify key genes, we screened genes in the “terpenoid skeleton biosynthesis” and “phenylpropanoid biosynthesis” pathways. The DEGs involved in the “terpenoid skeleton biosynthesis” pathway were clustered into two branches, with Branch 1 comprising the samples of WXY, FJ, WX, WS, TB, and NL and Branch 2 comprising the samples of GY and CK (Figure 7A). This division reflects the upregulation of key enzymes like geranylgeranyl diphosphate synthase and hydroxymethylglutaryl-CoA reductase in TB, GY, WS, and CK, which are indicative of an enhanced terpenoid biosynthesis capacity. Genes involved in the “phenylpropanoid biosynthesis pathway” were clustered into two branches based on their expression patterns, with samples from TB and GY having similar trends and being clustered into Branch 1 and the other samples with significant differences between their expression trends being clustered among different branches (Figure 7B). Comprising samples TB and GY, Branch 1 shows a coordinated expression of key genes, such as ‘Cinnamyl-alcohol dehydrogenase’ and ‘Phenylalanine ammonia-lyase’, essential for lignin synthesis and enhancing the plant structural integrity and stress response. Additionally, ‘Caffeoyl-CoA O-methyltransferase’, critical for flavonoid and lignan biosynthesis, also exhibited similar expression in these samples. Conversely, other samples displayed diverse expression profiles, with genes like ‘4-coumarate-CoA ligase’ and ‘Peroxidase’—involved in the initial steps of phenylpropanoid formation and defense against oxidative stress—forming distinct clusters. This expression landscape underscores the varied metabolic capabilities and adaptive responses across different biomass samples. The differential gene expression patterns in the terpenoid skeleton, “terpenoid skeleton biosynthesis,” and “phenylpropanoid biosynthesis” pathways were similar to the total gene expression patterns in different samples, suggesting that the differential genes in these two pathways may determine the differences and uniqueness of region samples. Terpenes are natural bioactive compounds found in many medicinal biomass sources. It has been reported that >20,000 terpene compounds have been discovered, including many important steroid hormone drugs and ginsenosides. The characteristic components of biomass, including imperialine, peimisine, peimine, and peiminine, are steroid alkaloids. Despite the large number of steroidal compounds, their synthesis mechanisms are similar, primarily being the cytoplasmic methyl valerate (MVA) and cytoplasmic methyl erythritol 4-phosphate (MEP) pathways. *HMGR* is a crucial regulatory factor in the synthesis of terpenoid frameworks. It catalyzes the conversion of 3-hydroxy-3-methylglutaryl CoA to methoxyvalerate, which is an irreversible rate-limiting reaction, with *HMGR* expression directly affecting steroidal saponin content. *HMGS* catalyzes the synthesis of acetylacetyl-CoA to form HMG-CoA, which is used for subsequent reactions in the synthesis of 3-hydroxy-3-methylglutaryl CoA synthesis. *TPS* is speculated to be located in chloroplasts, owing to its potential participation in terpene synthesis by engaging in the MEP pathway. The fragments per kilobase of transcript per million mapped reads (FPKM) values of the *HMGS*, *HMGR*, and *TPS* genes in different biomass samples are shown in Table S4. In each group, except for GY and TB, the FPKM values of the *HMGR* genes were higher than those of the WXY sample. The FPKM values of the *HMGS* genes in the TB and GY samples were 0, indicating significant differences compared to the other group samples. The FPKM value of the *TPS* gene in the TB sample was also 0, whereas the FPKM values of the *TPS* gene in the WS and GY samples were significantly lower than those in the other group samples.

### 3.4. qRT-PCR Validation of Candidate Genes and Determination of Four Alkaloid Components

The relative expression levels of the functional genes in eight biomass samples were calculated as shown in Figure S2. Among the DEGs, a significant difference was observed in the expression level of *HMGR*. The expression level of the *TPS* gene was highest in the FJ sample and lowest in the NL sample. There were no significant differences in the *HMGS* expression level among the samples. The qRT-PCR results of DEGs were consistent with transcriptome data, verifying the reliability of the transcriptome sequencing results. The contents of imperialine, peimisine, peimine, and peiminine in different biomass samples were measured (Table S5). Overall, the alkaloid content in the WX, WS, FJ, and CK samples was significantly higher compared with that of the other samples, followed by the NL and GY samples, whereas the TB sample had the relatively lowest content.

## 4. Discussion

### 4.1. Relationship Between the Alkaloid Content and the Candidate Gene Transcription Level

Research has shown the main quality determinant factor, such as the alkaloid content in the bulbs of *Fritillaria. spp* plants, is closely related to their varieties, growth status, and planting environment. Lu et al. [21] found *F. cirrhosae* accumulated higher levels of major steroidal alkaloids than *F. thunbergii*. Transcription factors involved in the biosynthesis of steroidal alkaloids may contribute to the differences in alkaloid content in the two variants. Harvesting times, processing methods [22], and even lights [23] were reported to affect the contents of alkaloids, further determining the quality of the bulbs of *F. cirrhosa*. However, there is relatively little research on the quality of *F. taipaiensis*, the changes in the biomass and alkaloid content of *F. taipaiensis* from different regions are not clear, and the key regulatory genes are also unknown. To investigate the relationship between the alkaloid content of different types of biomass samples and transcription levels of the *TPS*, *HMGR,* and *HMGS* candidate genes (Figure S3), correlation analyses between the alkaloid content of different biomass types and relative expression levels of the candidate genes were performed. The trend of the relative expression levels of the *HMGS* and *HMGR* genes is similar to the trend of the content of berberine and berberine B content, whereas the trend of the relative expression levels of the *TPS* genes is similar to the trend of the content of berberine A content. However, certain differences exist between the transcription levels of the three candidate genes and the trend of changes in the content of berberine and total alkaloids. The results indicate that the expression levels of different genes significantly differ in relation to the alkaloid content in various biomass types.

Pearson bivariate correlation analysis was applied to explore the correlation between the expression levels of *HMGS*, *HMGR,* and *TPS* genes and alkaloids in the mature bulbs of different biomass samples (Table S6). Only the expression level of *TPS* was highly positively correlated with Peimine content (r = 0.790, *p* = 0.02).

### 4.2. Analysis of the Components of F. taipaiensis Using UPLC-Q-TOF-MS/MS Technology

An analytical method for determining the chemical composition of different biomass samples using UPLC-Q-TOF-MS/MS (Agilent, Beijing, China) technology was established. To the best of our knowledge, an accumulation of different metabolites in biomass samples was analyzed for the first time. In total, 83 compounds were achieved in the LC–MS positive and negative ion modes, including alkaloids, nucleosides, organic and fatty acids, carbonyl compounds, esters, and sugars (mainly alkaloids). Otherwise, findings determined that alkaloids are the main bioactive components underlying the pharmacological properties of biomass samples [24], affirming the results of previous studies on the *Fritillaria* genus, including *F. cirrhosa* and *F.unibracteata* [25,26,27]. Therefore, this study greatly enriched the components of the biomass of alkaloid-related compounds.

### 4.3. Differences in the Medicinal Quality of F. taipaiensis from Different Regions

Using the relevant metabolite information, hierarchical cluster analysis, PCA, and OPLS-DA were conducted on different biomass samples, determining that different biomass samples from different regions exhibit significant differences in their chemical compositions, illustrating that the chemical composition and types of *F. taipaiensis* are significantly influenced by regional environmental factors. This result is consistent with previous studies on *F. thunbergii*, *Paris polyphylla* var. *yunnanensis*, and *Panax quinquefolius* [28,29,30]. Eight heterosteroidal alkaloid differential compounds were identified by OPLS-DA. UPLC–MS was used to quantitatively analyze the four main alkaloid differential metabolites of the different biomass samples. Thus, these compounds exhibited significant differences in content between the biomass samples. The contents of the WX, CK, and FJ samples were much higher than those in the other samples. However, the content in the WS sample was slightly lower than that in the WX, CK, and FJ samples. Thus, the medicinal quality of the bulbs of different biomass samples is habitat-dependent. However, there is limited in-depth research on the relationship between the changes in alkaloid content and quality of biomass samples.

### 4.4. DEGs in the Samples from Different Regions

The study of genetic resources has become a focus of recent research [21]. Recently, with the in-depth development of RNA-seq high-throughput sequencing technology, scholars have focused on the biosynthesis pathways of active ingredients and the functions of their related enzyme genes [31]. Transcriptome sequencing detected 359,529 transcripts and 174,827 unigenes, with 7035 unigenes co-annotated in seven databases (KOG, NT, KO, SwissProt, PFAM, and GO), accounting for 4.02% of the total unigenes. Overall, 468,036 DEGs were identified among different sample combinations, of which 242,857 were upregulated and 225,179 were downregulated, greatly enriching the transcriptomic and biological information of biomass samples. The DEGs between different samples in the KEGG database were mainly enriched in steroidal alkaloid synthesis, including terpenoid skeleton and phenylpropanoid biosyntheses, as well as stress response pathways, such as plant–pathogen interactions and plant hormone signal transduction. These findings provide important foundational data and research directions for revealing the biosynthesis mechanism of steroidal alkaloids and molecular mechanisms of biomass samples.

### 4.5. Discovery of Key Genes Involved in Differential Alkaloid Synthesis in F. taipaiensis from Different Regions

Previous studies have shown two main pathways for the synthesis of steroidal alkaloids: the MVA and MEP pathways [32]. Isopentenyl pyrophosphate is a common intermediate in both the MVA and MEP pathways. Isopentenyl pyrophosphate undergoes a series of enzymatic reactions that ultimately form steroid alkaloids [21]. Zhao et al. [10] found that in *F. cirrhosa*, the MEP pathway other than the MVA pathway was the main route for steroidal alkaloid biosynthesis using transcriptome analysis. We measured the alkaloid content of different biomass samples and found that the alkaloid content of the WXY samples was significantly higher than the GY and TB samples. Transcriptome KEGG annotation analysis revealed that in the terpenoid skeleton biosynthesis pathway, 21 DEGs were enriched between the GY and WXY samples while 28 DEGs were enriched between the TB and WXY samples. The key rate-limiting enzyme genes, such as *HMGS* and *HMGR*, in the terpenoid skeleton biosynthesis pathway were significantly downregulated in both the GY and TB samples, indicating that the downregulation of key genes in the terpenoid skeleton biosynthesis pathway is an important factor in alkaloid synthesis. *HMGS* and *HMGR* may be the key genes responsible for the differences in alkaloid synthesis across biomass samples, providing important data and insights for further exploration of the molecular mechanisms that affect the differences and uniqueness of various biomass samples.

### 4.6. Differences in the Gene Expression of F. taipaiensis from Different Regions

Transcriptome sequencing analysis of the bulbs from different biomass samples in this study showed that there were 240 DEGs (117 upregulated and 123 downregulated) between the WXY and WX samples and 163 DEGs (42 upregulated and 121 downregulated) between the WXY and NL samples. There were no significant differences in gene expression, which may be related to the introduction of NL-cultivated varieties originating from the WX sample. PCA showed that WXY grouped with the samples from WX, CK, FJ, WS, and NL. This indicates little difference in gene expression was observed between WXY and the other samples, possibly due to genetic basis factors. Zhou et al. reported similar results regarding the introduction and cultivation of Epimedium [33]. Therefore, further research is required to determine the magnitude of the effects of internal and external conditions on the metabolites.

### 4.7. Analysis of Systematic Signal Transduction Networks in F. taipaiensis from Different Regions

Biomass systems have complex and systematic signal transduction networks that actively respond to changes in internal factors and the external environment, thereby resisting environmental stress. In this network system, hormones are the main endogenous factors in plant stress responses, stress resistance regulation, and growth regulation [34]. KEGG enrichment analysis of WXY vs. GY samples in this study demonstrated that DEGs in WXY vs. TB samples were significantly enriched in the hormone signal transduction pathway. The plant–pathogen interaction pathway primarily acts on the complex, precise, and multilevel immune system formed during the long-term evolution of biomass [35]. The KEGG enrichment analysis results between the WXY and TB samples in this study showed significantly enriched differential genes in the plant–pathogen interaction pathway and differential genes, such as *CDPK*, *NOS,* and *WRKY25*, were upregulated in the WXY samples. It is speculated that there are certain differences in resistance to biological stress (pathogen infection) between the wild variety (WXY) and GY and TB samples.

### 4.8. Functional Speculation of Key Genes Involved in Alkaloid Synthesis in F. taipaiensis

With the continuous development of omics technology, multi-omics joint analysis is applied to analyze the complex biological mechanisms of biomass. In the joint analysis of the transcriptome and metabolome, the association analysis between DEGs in the transcriptome and differentially detected metabolites in the metabolome can be used to analyze changes at two levels, cause and effect; lock in key pathways related to metabolite changes; and construct a core regulatory network to reveal its internal laws [36]. Through transcriptome analysis, a large number of genes have been identified to play a regulatory role in alkaloid synthesis. Many transcription factors, including *bHLH*, *MYC*, *MYB*, and *WRKY*, regulate the alkaloid content by affecting the gene expression in the MVA pathway. *SQLE*, *CAS1*, *SMT1*, *SMO1*, *SMO2*, *SC5DL*, *DHCR7*, *DHCR24*, *CYP710A*, *3β-HSD*, *CYP90D2*, and *CYP374A6* genes were found to be differentially expressed in the bulb of *F. cirrhosa* [37].

Based on the differential metabolites of different biomass samples, the key genes involved in the synthesis pathways of the aforementioned substances were preliminarily validated, along with three key candidate genes: *HMGS*, *HMGR*, and *TPS*. The expression level of *TPS* was significantly positively correlated with the total alkaloid and peimine contents, whereas *HMGS* and *HMGR* had little effect on alkaloid content. The key genes play crucial roles in the upstream of the terpenoid biosynthesis pathway, including *HMGR*, farnesyl diphosphate synthase (*FPS*), squalene synthase (*SS*), and cycloartenol synthase (*CAS*), which have been well characterized, while the secondary metabolic pathways following the cycloartenol generation are rarely studied. Li et al. [13] revealed that *FcCYP450* of the predicted *CYP450* family, which was responsible for C-22, C-23, and C-26 hydroxylation in the steroidal alkaloid biosynthesis pathway, was related to the isosteroidal alkaloid biosynthesis. *TPS*s convert acyclic C5 to C20 cis- or trans-prenyl diphosphate intermediates into the C5-hemiterpenes, such as isoprene, C10-monoterpenoids, C15-sesquiterpenoids, or C20-diterpenoids [38]. Here, we found the expression level of *TPS* was highly correlated with alkaloid contents. It is speculated that *TPS* may be the main rate-limiting gene alkaloid biosynthesis, although further experimental evidence is needed. In the near future, using genetic engineering technology to enhance *TPS* gene expression may be a feasible approach to increase the amount of alkaloids in biomass samples.

In this study, three genes involved in alkaloid synthesis were selected for qRT-PCR. The upregulation and downregulation of expression trends in different biomass samples were consistent with the transcriptome FPKM values. This further proves the authenticity and reliability of the transcriptome sequencing results. Therefore, this study captured DEGs among different biomass samples through RNA high-throughput sequencing technology and systematically studied the genes involved in growth, development, and secondary metabolism as a whole, further elucidating the molecular mechanisms of active ingredient formation in biomass samples.

## 5. Conclusions

In summary, eight types of heterosteroidal alkaloids with significant differences among the *F. taipaiensis* biomass samples from different origins were selected, including peimisine, imperialine, peimine, peiminine, ebeinone, ebeiedine, ebeiedinone, and forticine. The *F. taipaiensis* samples can be clustered into two branches of DEGs: (1) NL, FJ, WX, CK, WS, and WXY; (2) TB and GY. The DEGs in the “terpenoid skeleton biosynthesis” and “phenylpropaneoid biosynthesis” pathways impacted the samples the most significantly. Eighty-three potential compounds were inferred in the biomass samples, including thirty alkaloids, six alkaloid glycosides, two fatty amides, nine nucleosides, one peptide, six fatty acids, six aromatic acids, ten lipids, one acid anhydride, three aromatic aldehydes, one aromatic alcohol, one aromatic ketone, one phenol, one fatty alcohol, and five sugar components. The alkaloid contents in samples WX, CK, and FJ were much higher than those in the other samples. The accumulation of active alkaloid components in different biomass samples was strongly correlated with the expression levels of catalytic enzyme genes involved in biosynthesis. Thus, *TPS* may be the main rate-limiting gene regulating alkaloid biosynthesis. This study reveals the biosynthesis and regulatory mechanisms of the main active ingredients and provides promising applications in the biomedical fields.

## Figures and Tables

**Table 1:** The information and codes of the different biomass samples.

Sample Group	Collection Site	Latitude and Longitude	Elevation/m
WXY	Yinchangping, Hongchiba, Wuxi County, Chongqing	31°37′46.30″ N/108°56′00.27″ E	2105
WX	Narrow Neck, Hongchiba Community, Wuxi County, Chongqing	31°37′32.05″ N/108°57′20.71″ E	1769
FJ	Meng Yuan Medicine Valley, Xicao Village, Xinglong Town, Fengjie County, Chongqing	30°45′47.47″ N/109°36′38.96″ E	1915
WS	Renziping, Lihe Village, Dangyang Township, Wushan County, Chongqing	30°46′07.21″ N/109°36′33.99″ E	1538
CK	Xiaolongtan, Sihe Village, Mingzhong Township, Chengkou County, Chongqing	31°41′14.08″ N/108°56′52.59″ E	1562
NL	Yangjiancao, Cuiyu Township, Ninglang County, Lijiang City, Yunnan Province	27°31′04.91″ N/100°35′39.14″ E	3372
TB	Tangkou Village, Zuitou Township, Taibai County, Baoji City, Shaanxi Province	34°03′25.82″ N/107°23′40.79″ E	1625
GY	Qinglin Village, Lijia Town, Chaotian District, Guangyuan City, Sichuan Province	32°35′10.06″ N/106°13′17.34″ E	1760

**Table 2:** qRT-PCR primer information.

Gene Name	KO Number	Forward Primer (5′→3′)	Reverse Primer (5′→3′)	Temperature (°C)
*HMGS*	K01641	CCAACCTTGCGAGCGAATA	TTGTAGGGAGAATGGAACACGA	59.60
*HMGR*	K00021	TGCGAGGCTCCTGGCTACT	CTTTGCTGGACCTGTTATACTTCA	60.70
*TPS*	K03527	AGGATCAAATCACGGGAGGC	ATCAATCCTCCAAGTCGCCC	59.82

## Data Availability

Raw data of this study can be download at https://www.ncbi.nlm.nih.gov/bioproject/PRJNA1036838 (accessed on 25 October 2024), with password: 7xfu.

## Supplementary Materials

The following supporting information can be downloaded at: https://www.mdpi.com/article/10.3390/metabo14110590/s1, Figure S1: Unigenes-Transcript length distribution in the transcriptome; Figure S2: qRT-PCR validation of the DEGs in biomass from different regions; Figure S3: The relationship between the total alkaloid content of biomass and the expression of *TPS*, *HMGR*, and *HMGS* genes; Table S1: Soil properties of the sample collection areas; Table S2: Mass spectrometry information of the compounds of biomass; Table S3: Transcriptome sequencing data quality analysis; Table S4: The contents of Chinese and Imperialine, Peimisine, Peimine, and Peiminine in biomass from different origins; Table S5: The correlation analyze between the expression levels of *HMGS*, *HMGR* and *TPS* genes and alkaloid content in mature bulbs of biomass from different regions; Table S6: The correlation analyze between the expression levels of *HMGS*, *HMGR* and *TPS* genes and alkaloid content in mature bulbs of biomass from different regions.
