# Supplementary material for: Isolation and Identification of Alkaloid Genes from the Biomass of Fritillaria taipaiensis P.Y. Li
Source: Metabolites. 2024 Oct 31;14(11):590. doi: 10.3390/metabo14110590 (PMC11596783; doi:10.3390/metabo14110590)
Supplement: Supplementary file 1 [file metabolites-14-00590-s001.zip › metabolites-3205142-supplementary.pdf]

Supporting information

Isolation and Identification of Alkaloid Genes from the Biomass of *Fritillaria taipaiensis*

P.Y. Li

Nong Zhou[1, 2], Chun-Mei Me[1], Fu-Gui Chen[1], Yu-Wei Zhao[1], Ming-Guo Ma[3,*], Wei-Dong Lia[1, *]

1 College of Pharmacy, Nanjing University of Chinese Medicine, Nanjing 210023, PR China;

2 College of Food and Biological Engineering, Chongqing Three Gorges University, Chongqing 404120, PR China;

3 College of Materials and Science and Technology, Beijing Forestry University, Beijing 100083, PR China.

* Correspondence: mg_ma@bjfu.edu.cn(M.M.); liweidong0801@163.com(W.L.) Tel.: +0086-10-62337250

*Corresponding author. Tel.: +0086-10-62337250. E-mail address: mg_ma@bjfu.edu.cn (M.-G. Ma), liweidong0801@163.com (W.-D. Li).

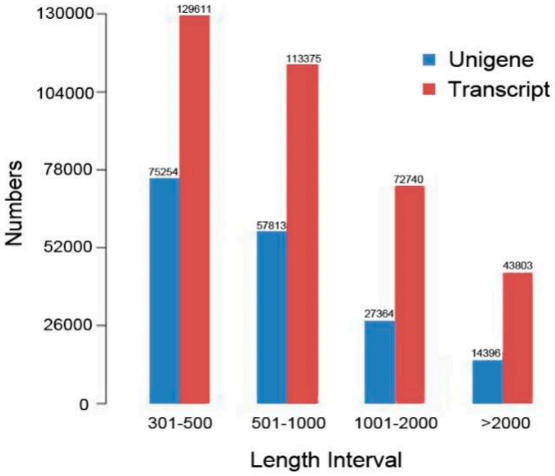

Figure S1. Unigenes-Transcript length distribution in the transcriptome.

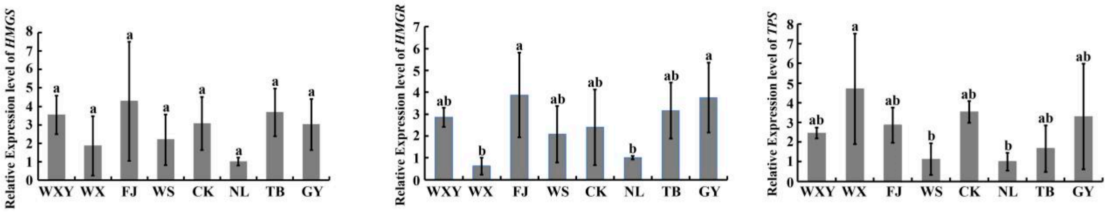

Figure S2. qRT-PCR validation of the DEGs in biomass from different regions.

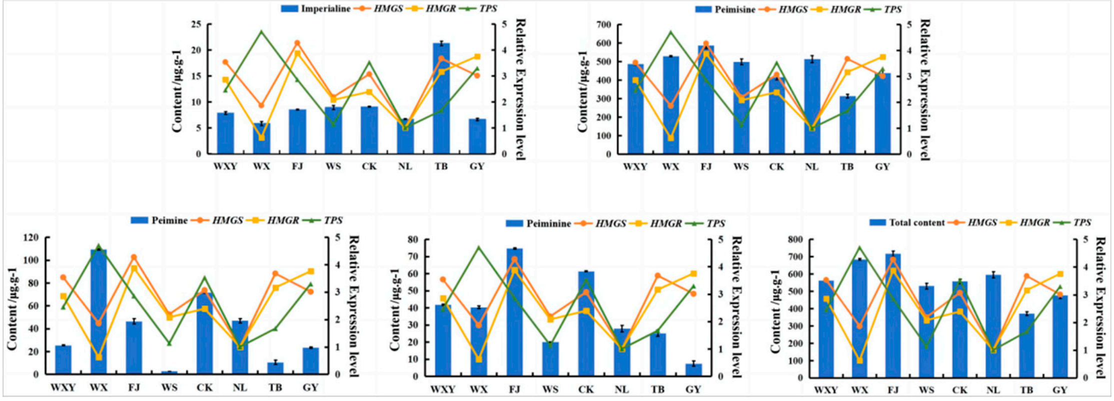

Figure S3. The relationship between the total alkaloid content of biomass and the expression of *TPS*, *HMGR*, and *HMGS* genes.

Table S1. Soil properties of the sample collection areas (n=3).

Notes: The different small letters labeled on the histogram indicate significant differences (P <0.05); the same small letter indicate no significant

No.	Organic matter /g.kg^{-1}	Total N /g.kg^{-1}	Total P /g.kg^{-1}	Total K /g.kg^{-1}	Available N /mg.kg^{-1}	Available P /mg.kg^{-1}	Available K /mg.kg^{-1}	pH
WXY	68.500±5.489a	4.737±0.052b	0.603±0.058f	9.295±0.086de	107.524±5.140e	4.232±0.697f	170.533±0.931c	
WX	65.671±4.301a	4.036±0.296c	1.173±0.079c	14.920±0.269a	76.349±5.706g	19.005±1.510a	123.941±17.600d	
FJ	36.740±1.174c	2.547±0.065e	1.419±0.046b	11.549±0.258b	77.693±6.711g	11.688±1.912d	273.722±14.124a	
WS	26.105±0.712d	1.920±0.213f	0.960±0.064d	8.830±0.281e	89.541±8.447f	9.567±1.453e	240.130±9.690b	
CK	48.716±3.529b	2.981±0.141d	0.661±0.054f	9.413±0.330d	146.079±9.053b	2.150±0.505g	77.972±8.260e	
NL	13.526±2.798e	7.945±0.199a	2.183±0.073a	14.457±0.351a	118.582±4.048d	9.391±1.705e	282.582±9.342a	
TB	25.628±1.551d	1.566±0.040g	0.784±0.060e	6.929±0.164f	150.741±12.444a	15.752±1.553b	163.565±6.069c	
GY	27.096±2.019d	1.899±0.057f	1.387±0.049b	10.115±0.445c	135.433±4.697c	13.518±0.463c	163.346±8.011c	

differences (P >0.05); in the following figures, the small letters also mean the significant differences at the 0.05 level.

Table S2. Mass spectrometry information of the compounds of biomass.

Serial number	tR/min	Molecular formula	Mode	Theoretical value m/z	Measured value m/z	Errors /10-6	Identification of components	Component type
1	4.73	C8H10N4O2	[M+H]+	195.0877	195.0873	-1.6	caffeine	Alkaloid
2	5.77	C27H43NO4	[M+H]+	446.3265	446.3264	-0.2	imperialine-β-N-oxide	Alkaloid
3	6.08	C33H51NO8	[M+H]+	590.3687	590.3697	1.6	peimisine-3-O-β-D-glucopyranoside	Alkaloid glycoside
4	6.40	C27H39NO4	[M+H]+	442.2952	442.296	1.9	delavidine	Alkaloid
5	6.48	C27H47NO4	[M+H]+	450.3578	450.3572	-1.2	cirrhosinine A	Alkaloid
6	6.93	C33H53NO8	[M+H]+	592.3844	592.3854	1.7	sipeimine3-O-β-D-glucoside	Alkaloid glycoside
7	6.93	C33H53NO8	[M+H]+	592.3844	592.3854	1.7	imperialine-3-O-β-D-glucoside	Alkaloid glycoside
8	6.98	C27H45NO4	[M+H]+	448.3421	448.3426	1.1	cirrhosinine B	Alkaloid
9	6.98	C27H45NO4	[M+H]+	448.3421	448.3426	1.1	isoverticine-β-N-oxide	Alkaloid
10	7.15	C27H43NO3	[M+H]+	430.3316	430.3318	0.6	imperialine	Alkaloid
11	7.15	C27H43NO3	[M+H]+	430.3316	430.3318	0.6	delafrinone	Alkaloid
12	7.15	C27H43NO3	[M+H]+	430.3316	430.3318	0.6	peiminine	Alkaloid
13	7.24	C27H41NO3	[M+H]+	428.3159	428.3174	3.5	peimisine	Alkaloid
14	7.24	C27H41NO3	[M+H]+	428.3159	428.3174	3.5	songbeisine	Alkaloid
15	7.24	C27H41NO3	[M+H]+	428.3159	428.3174	3.5	songbeinine	Alkaloid
16	7.44	C27H45NO3	[M+H]+	432.3472	432.348	1.9	peimine	Alkaloid
17	7.44	C27H45NO3	[M+H]+	432.3472	432.348	1.9	isoverticine	Alkaloid
18	7.44	C27H45NO3	[M+H]+	432.3472	432.348	1.9	delafrine	Alkaloid
19	7.61	C33H53NO7	[M+H]+	576.3895	576.3896	0.1	Puqiedinone-3-O-β-D-glucopyranoside	Alkaloid glycoside
20	7.93	C33H53NO7	[M+H]+	576.3895	576.3904	1.5	yibeinoside B	Alkaloid glycoside
21	7.93	C33H53NO7	[M+H]+	576.3895	576.3904	1.5	yibeinoside A	Alkaloid glycoside
22	8.66	C27H43NO2	[M+H]+	414.3367	414.3373	1.6	delavinone	Alkaloid
23	8.66	C27H43NO2	[M+H]+	414.3367	414.3373	1.6	chuanbeinone	Alkaloid
24	8.66	C27H43NO2	[M+H]+	414.3367	414.3373	1.6	ebeiedinone	Alkaloid
25	8.66	C27H43NO2	[M+H]+	414.3367	414.3373	1.6	puqiedinone	Alkaloid
26	8.66	C27H43NO2	[M+H]+	414.3367	414.3373	1.6	songbeinone	Alkaloid
27	8.78	C27H45NO2	[M+H]+	416.3523	416.3526	0.8	forticine	Alkaloid
28	8.97	C27H41NO2	[M+H]+	412.321	412.3219	2.1	Ebeinone	Alkaloid
29	9.47	C27H45NO2	[M+H]+	416.3523	416.3533	2.5	isodelavine	Alkaloid
30	9.47	C27H45NO2	[M+H]+	416.3523	416.3533	2.5	isoforticine	Alkaloid
31	9.47	C27H45NO2	[M+H]+	416.3523	416.3533	2.5	petilidine	Alkaloid
32	9.47	C27H45NO2	[M+H]+	416.3523	416.3533	2.5	Puqiedine	Alkaloid
33	9.47	C27H45NO2	[M+H]+	416.3523	416.3533	2.5	ebeiedine	Alkaloid
34	9.47	C27H45NO2	[M+H]+	416.3523	416.3533	2.5	delavine	Alkaloid

35	10.72	C27H43NO	[M+H]+	398.3417	398.3424	1.7	Solanidine	Alkaloid
36	10.77	C27H45NO	[M+H]+	400.3574	400.3573	-0.3	demissidine	Alkaloid
37	26.81	C22H43NO	[M+H]+	338.3417	338.3427	2.9	cis-13-docosenoamide	Fatty amide
38	24.85	C6H11NO	[M+H]+	114.0913	114.0913	0	caprolactam	Fatty amide
39	0.70	C9H13N3O5	[M-H]-	242.0782	242.0783	0.1	cytidine	Nucleoside
40	0.72	C10H13N5O3	[M+H]+	252.1091	252.109	-0.7	2'-deoxyadenosine	Nucleoside
41	0.74	C10H12N4O5	[M-H]-	267.0735	267.0738	1.0	inosine	Nucleoside
42	1.01	C4H4N2O2	[M+H]+	113.0346	113.0345	-0.5	uracil	Nucleoside
43	1.08	C9H12N2O6	[M+H]+	245.0768	245.0767	-0.7	uridine	Nucleoside
44	1.16	C10H13N5O4	[M+H]+	268.104	268.1047	2.4	adenosine	Nucleoside
45	1.22	C10H13N5O5	[M+H]+	284.099	284.0991	0.4	guanosine	Nucleoside
46	1.22	C5H5N5O	[M+H]+	152.0567	152.0567	0.1	guanine	Nucleoside
47	13.07	C11H18N2O2	[M+H]+	211.1441	211.1445	1.8	cyclo-(pro-leu)	Peptide
48	28.45	C5H5N5	[M+H]+	136.0618	136.0616	-1.1	adenine	Nucleoside
49	17.24	C18H30O2	[M+H]+	279.2319	279.2326	2.7	linolenic acid	Fatty acid
50	22.6	C16H32O2	[M+H]+	257.2475	257.2483	3.2	Palmitic acid	Fatty acid
51	22.82	C18H32O2	[M+H]+	281.2475	281.2483	2.9	linoleic acid	Fatty acid
52	24.44	C18H34O2	[M-H]-	281.2486	281.2486	-0.2	oleic acid	Fatty acid
53	26.19	C18H36O2	[M-H]-	283.2643	283.2642	-0.2	octadecanoic acid	Fatty acid
54	1.09	C9H8O3	[M+H]+	165.0546	165.0547	0.7	E-p-hydroxycinnamic acid	Organic acid
55	1.09	C9H8O3	[M+H]+	165.0546	165.0547	0.7	Cis-4-coumaric acid	Organic acid
56	1.09	C9H8O3	[M+H]+	165.0546	165.0547	0.7	p-coumaric acid	Organic acid
57	5.78	C12H14O5	[M+H]+	239.0914	239.0924	4.4	E-3,4,5-trimethoxycinnamic acid	Organic acid
58	6.52	C10H10O4	[M-H]-	193.0506	193.0505	-0.5	ferulic acid	Organic acid
59	21.69	C14H28O2	[M-H]-	227.2017	227.202	1.4	Tetradecanoic acid	Fatty acid
60	22.40	C10H10O3	[M+H]+	179.0703	179.0701	-1.1	E-p-methoxycinnamic acid	Organic acid
61	0.06	C5H10O2	[M+H]+	103.0754	103.0749	-4.0	Formic acid butyl ester	Terpenoid
62	1.09	C5H4O3	[M-H]-	111.0088	111.0085	-2.2	Itaconic anhydride	Acid anhydride
63	4.69	C6H10O3	[M-H]-	129.0557	129.0554	-2.6	DL-pantolactone	Terpenoid
64	6.33	C8H8O3	[M+H]+	153.0546	153.0546	-0.5	Vanillin	Aromatic aldehyde
65	8.82	C19H38O2	[M+H]+	299.2945	299.2946	0.4	methyl stearate	Terpenoid
66	13.47	C8H8O	[M+H]+	121.0648	121.0649	0.6	phenylacetaldehyde	Aromatic aldehyde
67	18.06	C11H12O	[M+H]+	161.0961	161.0964	1.9	2-cyclopenten-1-ol，1-phenyl-	Aromatic alcohol
68	18.06	C11H12O	[M+H]+	161.0961	161.0964	1.9	3-methyl-4-phenylbut-3-en-2-one	Aromatic ketone

69	18.90	C16H22O4	[M+H]+	279.1591	279.1601	3.6	Diisobutyl phthalate	Terpenoid
70	18.95	C14H20O	[M+H]+	207.1743	207.1745	0.6	2,4-di-tert-butylphenol	Phenol
71	21.28	C21H38O4	[M+H]+	355.2843	355.2845	0.6	1-monolinolein	Terpenoid
72	22.40	C10H10O3	[M+H]+	179.0703	179.0701	-1.1	E-p-hydroxycinnamic acid methyl ester	Terpenoid
73	22.59	C19H38O4	[M+H]+	331.2843	331.2852	2.7	2-monopalmitin	Terpenoid
74	22.81	C7H6O2	[M-H]-	121.0295	121.0289	-4.6	4-hydroxy benzaldehyde	Aromatic aldehyde
75	24.97	C21H42O4	[M+H]+	359.3156	359.316	1.2	glyceryl monostearate	Terpenoid
76	26.07	C19H34O2	[M+H]+	295.2632	295.2638	2.3	stearolic acid methyl ester	Terpenoid
77	27.82	C6H12O2	[M-H]-	115.0765	115.0766	0.9	Acetic acid butyl ester	Terpenoid
78	29.88	C20H42O	[M-H]-	297.3163	297.3176	4.5	1-eicosanol	Fatty alcohol
79	0.71	C6H14O6	[M-H]-	181.0718	181.0719	0.5	L-rhamnose monohydrate	Carbohydrate
80	0.74	C12H22O11	[M+H]+	343.1235	343.1242	2.2	D-sucrose	Carbohydrate
81	0.75	C6H12O6	[M+H]+	181.0707	181.0701	-3.1	D-mannose	Carbohydrate
82	0.75	C6H12O6	[M+H]+	181.0707	181.0701	-3.1	D-galactose	Carbohydrate
83	2.38	C5H10O5	[M+H]+	151.0601	151.0604	2.0	D-xylose	Carbohydrate

Table S3. Transcriptome sequencing data quality analysis.

Sample name	Raw reads/bp	Clean reads/bp	Clean data	Q20/%	Q30/%	GC content/%
WXY1	22 436 745	21 995 552	6.6	97.8	93.76	50.37
WXY2	23 135 470	22 153 080	6.6	97.98	94.07	48.63
WXY3	20 595 090	19 848 804	6.0	97.78	93.83	49.97
WX1	23 004 743	22 352 574	6.7	97.74	93.82	51.13
WX2	21 731 883	21 022 304	6.3	97.85	94.03	50.53
WX3	22 398 907	21 734 075	6.5	97.77	93.81	50.60
FJ1	23 586 898	22 863 240	6.9	97.9	94.2	50.89
FJ2	23 881 777	23 375 492	7.0	97.93	94.28	51.19
FJ3	22 259 375	21 691 613	6.5	97.97	94.3	50.8
WS1	21 417 182	20 691 620	6.2	97.78	93.89	50.92
WS2	20 997 405	20 383 021	6.1	98.21	94.68	47.04
WS3	21 376 887	20 513 203	6.2	97.92	94.16	50.43
CK1	22 158 566	21 446 346	6.4	97.6	93.54	50.87
CK2	23 332 491	22 278 480	6.7	97.58	93.28	49.03
CK3	22 963 208	21 691 020	6.5	97.7	93.71	50.25
NL1	22 659 240	21 647 206	6.5	97.86	94.04	50.78
NL2	22 994 330	22 063 092	6.6	97.89	94.23	50.87
NL3	22 027 351	21 360 478	6.4	97.55	93.28	50.7
TB1	22 676 182	21 616 388	6.5	98.13	94.57	49.26
TB2	23 087 692	22 681 362	6.8	97.94	94.25	50.93
TB3	23 286 212	22 793 281	6.8	97.89	94.18	50.69
GY1	21 235 596	20 884 258	6.3	97.83	94.03	51.22
GY2	21 369 998	21 025 504	6.3	97.62	93.51	50.53
GY3	21 778 445	21 457 966	6.4	97.49	93.19	50.97

Table S4. The contents of Chinese and Imperialine, Peimisine, Peimine, and Peiminine in biomass from different origins (μg.g^{-1}, n=3).

Content / Sample	**Imperialine**	**Peimisine**	**Peimine**	**Peiminine**	**Total amount**
WXY	7.874±0.247c	488.048±14.255c	25.482±0.663d	41.833±1.557c	563.237±16.106cd
WX	5.870±0.082e	529.411±15.432b	109.396±2.265a	40.399±0.345c	685.076±16.112b
FJ	8.546±0.087bc	586.728±19.080a	46.545±1.892c	74.761±1.979a	716.580±18.797a
WS	8.970±0.392b	498.691±15.776bc	2.647±0.028f	19.982±0.246e	530.290±15.851d
CK	9.110±0.415b	415.384±10.287d	71.503±1.986b	61.355±1.796b	557.352±11.265cd
NL	6.718±0.249d	514.088±6.290b	46.986±0.331c	27.934±0.418d	595.727±6.135c
TB	21.302±0.372a	313.889±4.332e	10.623±0.441e	25.190±0.693d	371.004±5.191f
GY	6.675±0.080d	438.001±12.562d	23.484±0.522d	7.481±0.258f	475.641±13.303e

Table S5. The correlation analyze between the expression levels of *HMGS*, *HMGR* and *TPS* genes and alkaloid content in mature bulbs of biomass from different regions.

	**HMGS**	**HMGR**	**TPS**	**Imperialine**	**Peimisine**	**Peimine**	**Peiminine**	**Total content**
HMGS	1.000							
HMGR	0.765**	1.000						
TPS	0.311	0.213	1.000					
Imperialine	0.221	0.225	-0.217	1.000				
Peimisine	-0.051	-0.114	0.062	-0.760**	1.000			
Peimine	-0.141	-0.372	0.476*	-0.447*	0.351	1.000		
Peiminine	0.216	0.066	0.212	-0.113	0.402	0.473*	1.000	
Total content	-0.030	-0.181	0.230	-0.696**	0.915**	0.657**	0.649**	1.000

Table S6. The correlation analyze between the expression levels of *HMGS*, *HMGR* and *TPS* genes and alkaloid content in mature bulbs of biomass from different regions.

		**HMGR**	**TPS**	**HMGS**	**Imperialine**	**Peimisine**	**Peiminine**	**Peimine**
HMGR	Pearson correlation	1.000	-0.088	0.902**	-0.263	-0.177	0.27	-0.543
	Significance (two-sided)		0.836	0.002	0.53	0.674	0.517	0.164
TPS	Pearson correlation	-0.088	1.000	0.17	0.256	0.35	0.422	0.790*
	Significance (two-sided)	0.836		0.687	0.541	0.395	0.298	0.02
HMGS	Pearson correlation	0.902**	0.170	1.000	-0.028	-0.177	0.517	-0.291
	Significance (two-sided)	0.002	0.687		0.948	0.675	0.189	0.484
Imperialine	Pearson correlation	-0.263	0.256	-0.028	1.000	0.422	0.793*	0.548
	Significance (two-sided)	0.530	0.541	0.948		0.298	0.019	0.160
Peimisine	Pearson correlation	-0.177	0.350	-0.177	0.422	1.000	0.439	0.491
	Significance (two-sided)	0.674	0.395	0.675	0.298		0.276	0.217
Peiminine	Pearson correlation	0.270	0.422	0.517	0.793*	0.439	1.000	0.428
	Significance (two-sided)	0.517	0.298	0.189	0.019	0.276		0.291
Peimine	Pearson correlation	-0.543	0.790*	-0.291	0.548	0.491	0.428	1.000
	Significance (two-sided)	0.164	0.020	0.484	0.160	0.217	0.291	
